# Recurrent *Streptococcus Pneumoniae 23 F m*eningitis due to cerebrospinal fluid leakage from the ear cannel: a case report

**DOI:** 10.1186/s12887-015-0509-2

**Published:** 2015-11-25

**Authors:** Yu-Cheng Li, Chun-Yu Chen, Kang-Hsi Wu, Huang-Tsung Kuo, Han-Ping Wu

**Affiliations:** Department of Pediatrics, Taichung Tzuchi Hospital, the Buddhist Medical Foundation, Taichung, Taiwan R.O.C; Division of Emergency Medicine, Department of Pediatrics, Changhua Christian Hospital, Changhua, Taiwan R.O.C; School of medicine, Chung Shan Medical University, Taichung, Taiwan ROC; School of Chinese Medicine, China Medical University, Taichung, Taiwan ROC; Department of Hemato-oncology, Children’s Hospital, China Medical University Hospital, China Medical University, Taichung, Taiwan ROC; School of Medicine, China Medical University, Taichung, Taiwan R.O.C.; Division of Developmental and Behavioral Pediatrics, Children’s Hospital, China Medical University, Taichung, Taiwan ROC; Division of Pediatric General Medicine, Department of Pediatrics, Chang Gung Memorial Hospital at Linko, Kweishan, Taoyuan, Taiwan R.O.C; College of Medicine, Chang Gung University, Taoyuan, Taiwan R.O.C

**Keywords:** Streptococcus pneumoniae, Recurrent, Meningitis, Cerebrospinal fluid

## Abstract

**Background:**

Bacterial meningitis is a medical emergency, and immediate diagnostic steps must be taken to establish the specific cause. Recurrence of bacterial meningitis in children is not only potentially life-threatening, but also involves or induces psychological trauma to the patients through repeated hospitalization with many invasive investigations.

**Case presentation:**

A 6-year-old boy was diagnosed with recurrent bacterial meningitis caused by *Streptococcus Pneumonia 23 F*. He had received serial imaging studies for identifying the cause. The initial sinus computed tomography (CT) also showed sinusitis without bony defect of sinus. However, after performing nuclear scan, the results showed cerebrospinal fluid (CSF) leaked originating from the right petrooccpital region into the middle ear. Subsequent high resolution CT (HRCT) reports showed focal enlargement of the right facial nerve canal, erosion of the bony canal at geniculate ganglion and tympanic segment with tiny high-density spots. The reconstruction HRCT showed multiple bony defects at temporal bone. The magnetic resonance imaging revealed multifocal bony destruction with CSF collection in the right petrous ridge, carotid canal and jugular foramen. Eventually, CSF leakage to the right middle ear was confirmed and this could be the cause of the recurrent bacteria meningitis in this patient.

**Conclusion:**

Although recurrent bacterial meningitis in childhood is not common, this case report illustrates that recurrence of meningitis within a short period should be considered as cause of underline immunologic or anatomic defect.

## Background

Bacterial meningitis is a medical emergency, and immediate diagnostic steps must be taken to establish the specific cause so that appropriate antimicrobial therapy can be initiated [1, 2]. The mortality rate of untreated bacterial meningitis approaches 100 % and, even with optimal therapy, morbidity and mortality may occur [2, 3]. Recurrence of bacterial meningitis in children may be caused by many reasons from cranial or dural anatomic defect and immumity deficiency [4]. Bacteria migration, along congenital or acquired pathways from the skull or spinal dural defects should be taken into consideration when children had recurrent bacteria meningitis [5]. However, symptoms and signs of cerebrospinal fluid (CSF) rhinorrhea or otorrhea are difficult to find in such patients [6]. The CSF leakage caused by traumatic injury is common, while leakage caused by congenital bony abnormality is rarely reported. Here we present the case of a 6-year-old boy with repeated bacterial meningitis within 6 months and further imaging exanimations finally proved the cause of CSF leakage originating from the right petrooccpital region into the middle ear.

## Case presentation

**Fig. 1:**
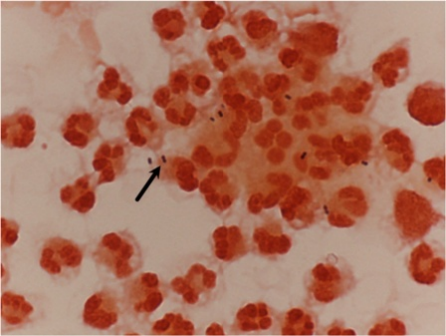
Gram stain of the CSF showed *Streptococcus Pneumoniae* (*black arrow*) in the patient

The 6-year-old boy complained of nausea, vomiting and headache for one week. He received medical treatment at local medical clinics initially, but his condition still persisted without improvement. Progressed symptoms and fever were also noted after initial medical treatment, and, he was transferred to our emergency department (ED) for further evaluation. At the ED, the previous history of the patient was obtained from his family. This boy had experienced one earlier episode of AOM in his young-infant stage and experienced a single episode of acute sinusitis about 2 months prior to admission. Moreover, no any history of skull trauma was noted before admission. However, the physical examinations revealed general appearance as lethargy and neck stiffness with positive meningitis signs (Brudzinski’s sign and Kerning sign). After admission, blood was sampled for complete blood count (CBC) with differential count (DC) analysis, biochemistry, glucose levels, and blood culture. Immediately lumbar puncture with CSF survey (CSF analysis, bacterial culture, virus culture and CSF biochemistry test) was also performed. The blood laboratory tests showed leukocytosis with shift to the left (white blood cell (WBC) count: 29190/mm^3^, and bands: 4 %), and the results of CSF analysis revealed WBC count as 3240/uL with predominant neutrophils as 91 %, glucose levels as 55 mg/dL, and total protein levels as 160.5 mg/dL. Moreover, the gram stain of CSF showed *Sptreptococcus Pneumoniae* (Fig. 1), and antibiotics with vancomycin and cefotaxime were given immediately. The cultures of CSF and blood both showed *Sptreptococcus Pneumoniae 23 F*. Based on the report of the sensitivity to antibiotics in the strain of *23 F*, vancomycin was useful and given continuously for 14 days. To trace back his past history, about 6 months ago, this pediatric patient suffered from bacterial meningitis, and was admitted for survey and treatments. The CSF gram stain showed *Sptreptococcus Pneumoniae*. Both CSF and blood cultures also showed *Sptreptococcus Pneumoniae 23 F*. After complete antimicrobial treatment with vancomycin for 14 days, he was discharged home without complication.

**Fig. 2:**
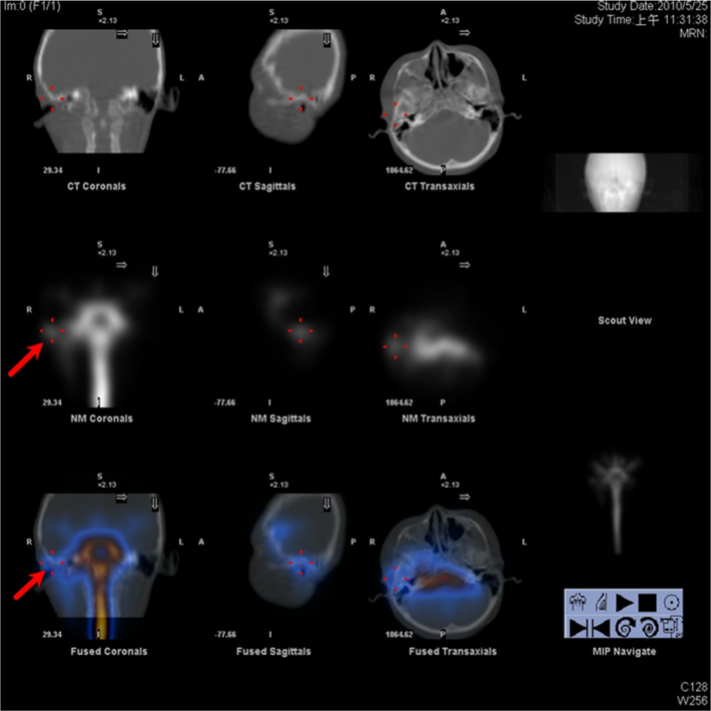
Radioisotope cisternography showed CSF leak into right side middle ear area (*red arrow*)

**Fig. 3:**
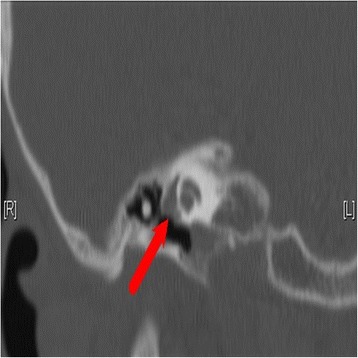
HRCT of the right side ear showed enlargement to facial nerve cannel (*red arrow*)

**Fig. 4:**
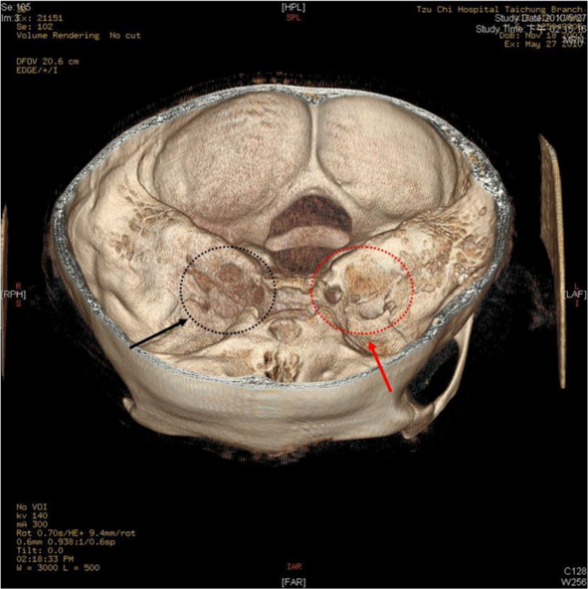
Reconstruction in brain HRCT showed multiple bony destructions at the right side (*black arrow*) compared to the left side (*red arrow*)

**Fig. 5:**
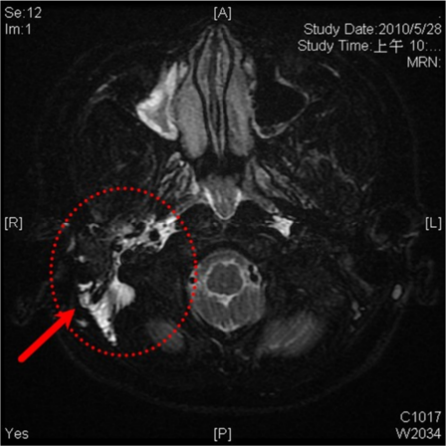
MRI showed CSF accumulation at right middle ear

To further survey the cause of recurrent bacteria meningitis in such short period, we analyzed immunological functions of this boy, including complements and various immunoglobulins. However, the results showed normal immunity. According to the previous history of recurrent sinusitis for several weeks, we suspected that recurrent meningitis may be due to a bony defect caused by chronic sinusitis. Sinus computed tomography (CT) was performed but only right side maxillary sinusitis was noted without any bony defect. Moreover, nuclear scan was arranged and performed for studying CSF leakage. Notably, the results showed CSF leaked originating from the right petrooccpital region into the middle ear (Fig. 2). Subsequent high resolution CT (HRCT) and magnetic resonance imaging (MRI) of bilateral ears were both carried out. The HRCT reports showed focal enlargement of the right facial nerve canal, erosion of the bony canal at geniculate ganglion and tympanic segment with tiny high-density spots (Fig. 3) and the reconstruction HRCT showed multiple bony defect at petrous part of temporal bone (Fig. 4). The MRI reports revealed multifocal bony destruction with CSF collection in the right petrous ridge (near the Meckel cave and facial nerve canal at geniculate body ganglion region), carotid canal and jugular foramen (Fig. 5). Eventually, CSF leakage to the right middle ear was confirmed and this may explain the cause of the recurrent bacteria meningitis in this boy. Further surgical approach for bony defect was suggested, but his family refused and asked for medical treatments. Therefore, after complete antimicrobial treatments with vancomycin for 14 days, this patient was discharged home, and received conjugated streptococcus pneumoniae vaccination (Prevenar 7) by self-payment, which is not included in the program of our national schedule vaccination at that time.

## Discussion

Recurrence of bacterial meningitis in children is not only potentially life-threatening, but also involves or induces psychological trauma to the patients through repeated hospitalization with many invasive investigations. In our case report, this patient had suffered from bacteria meningitis twice, and required repeated hospitalization for invasive CSF survey and for managements of infectious emergency. This situation did suffer very much for the patient and his family. Therefore, to avoid repeated meningitis again is essential for this patient and to understand why recurrence of bacterial meningitis occurred is also important for primary clinicians. Clinically, it is reasonable for primary clinicians to survey for immune deficiency or CSF leakage caused by defect of anatomy [6–11]. In addition, the bacteria specificity could provide some informative clues: Based on some investigations, pneumoccocus or hemophilus may suggest cranial dural defects, E. coli or other gram negative bacillus may suggest spinal dural defects, and meningococcus may suggest immunologic deficiency of the patient [3–5].

Moreover, spontaneous cerebrospinal fistula could be difficult for clinicians to make the diagnosis and only revealed recurrent attacks of meningitis. Recurrent meningitis may occur in 92 % of such fistulas which indicates the importance of accurate diagnosis and appropriate treatments for CSF leakage [7]. Recurrent meningitis, clear otorrhea, or rhinorrhea are signs requiring several investigations of the temporal bone. When the ear drum is intact, CSF passes down the eustachian tube and may result in rhinorrhea. If the tympanic membrane is perforated, either spontaneously or after myringotomy, otorrhea may occur. Some case reports have reported that congenital CSF leakage may present as serous otitis media and be revealed at the time of myringotomy [12]. Also, CT scan involving 1-mm sections in coronal and axial planes of the temporal bone is certainly the most precise and reliable method available [13, 14]. In our case report, the initial CT scan could not find out the leakage This may be due to the difficult in identifying the right location of CSF leakage by routine head or brain CT scan which is too broad to image the otic capsule, ossicles, and facial nerve accurately. Furthermore, the coronal images are usually reconstructions, which provide significantly less detail than the directly-obtained coronal image.

To test for CSF leakage, clinician may test the ear or nose drainage for beta-2 transferrin, a desialylated form of the protein transferring, which is almost exclusively found only in CSF [15, 16]. Therefore, to localization of the fistula may require diagnostic imaging studies [17]. Nuclear medicine examination (Radioisotope cisternography) or fluorescein dye study via lumbar puncture should be considered to identify the location of leakage [18]. In our case report, radioisotope cisternography combined with HRCT (1-mm section) and MRI appeared helpful to identify the location. From this case report, we found that recurrent bacteria meningitis is critical and should be prompt a search for an underline immunologic or anatomic defect. CSF leakage is common to cause misdiagnosis or failure to make a timely early diagnosis, which means that suitable treatment may be delayed. Better knowledge of the possible sites and pathways of fistulas (even rare ones) is necessary. The different pathways of spontaneous CSF leakage should be clearly understood and carefully examined by the radiologists and primary clinicians. Congenital inner ear malformation is an uncommon fistula route, which can be misdiagnosed even regular CT (usually cut every 5 mm) is performed without performed high resolution CT (usually cut every 1 mm). The treatment for this congenital fistula is based on filling of the bone pathway, which can be repaired with biometerials.

## Conclusions

Although recurrent bacterial meningitis in childhood is not common, this clinical condition remains a neurological emergency for primary care physicians. This case illustrates that recurrence of meningitis within a short period should be considered as cause of underline immunologic or anatomic defect.

## Consent

Written informed consent was obtained from the patient’s parents for publication of this Case report and any accompanying images. A copy of the written consents is available for review by the Editor of this journal.

## Abbreviations

ED: Emergency department
CSF: Cerebrospinal fluid
WBC: White blood cell
CT: Computed tomography
HRCT: High resolution computed tomography
